# Loss of TXNIP enhances peritoneal metastasis and can be abrogated by dual TORC1/2 inhibition

**DOI:** 10.18632/oncotarget.26281

**Published:** 2018-11-02

**Authors:** Douglas Spaeth-Cook, Mark Burch, Robin Belton, Bryce Demoret, Nicholas Grosenbacher, Jason David, Colin Stets, David Cohen, Reena Shakya, John L. Hays, James L. Chen

**Affiliations:** ^1^ Department of Biomedical Informatics, Division of Bioinformatics, The Ohio State University, Columbus, OH 43210, USA; ^2^ Department of Biostatistics, The Ohio State University, Columbus, OH 43210, USA; ^3^ Department of Internal Medicine, Division of Medical Oncology, The Ohio State University, Columbus, OH 43210, USA; ^4^ Department of Pathology, Anatomic Pathology Division, The Ohio State University, Columbus, OH 43210, USA; ^5^ James Comprehensive Cancer Center, The Ohio State University, Columbus, OH 43210, USA; ^6^ Department of Obstetrics and Gynecology, Division of Gynecologic Oncology, The Ohio State University, Columbus, 43210 OH, USA

**Keywords:** carcinomatosis, sarcomatosis, TXNIP, peritoneal disease

## Abstract

Peritoneal metastasis (PM) is a debilitating consequence of multiple cancers. As cancer cells lose tonic signaling related to attachment dependence, critical morphologic shifts result in alteration of the transcriptome. Identifying key genes associated with this transformation may lead to targeted therapies for this devastating complication. TC71, CHLA9, PANC1, YOU and HEYA8 cell lines were grown as tumor spheroids in polyHEMA coated plates. Temporal profiling of transcriptomic alterations over 72 hrs was used to develop a comprehensive PM model. We identified transcriptomic outliers using Gaussian mixtures model clustering to identify drivers of spheroid formation. Outliers were validated in The Cancer Genome Atlas (TCGA) and an ovarian tissue microarray (TMA) and by modulation in ovarian cancer models *in vitro* and in peritoneal xenograft models. Outlier analysis of PM genes identified the gene TXNIP and the TORC signaling as central to PM. Ovarian cancer spheroids isolated from patient ascites had significantly higher TXNIP than their attached counterparts (*p* = 0.047). TXNIP levels predicted progression-free (log-rank *p* = 0.026) survival in stage 1/2 ovarian cancer and overall survival (log rank *p* = 0.047) in stage 3/4 ovarian cancer. *In vitro*, TXNIP silencing was associated with increased mTOR signaling and enhanced spheroid development which could be overcome by TAK228, a TORC1/2 inhibitor. Similarly, *in vivo* peritoneal xenograft models of carcinomatosis were prevented by TAK228. PM is driven by TXNIP-associated TORC1/2 signaling. This work provides the first evidence that TORC1/2 inhibition may prevent PM.

## INTRODUCTION

There is an unmet need to develop effective and novel treatment strategies to prevent and treat peritoneal metastasis (PM). Multiple cancers, including gastrointestinal, gynecological and sarcomatous malignancies, have the potential to grow and disseminate throughout the peritoneal cavity [1, 2]. Up to 50% of gastric cancer, >80% of ovarian cancer, 10–20% of pancreatic cancers, and 20% of sarcomas can present with peritoneal surface dissemination or isolated peritoneal recurrence [3]. PM is the second most common site of recurrence in colorectal cancer [4]. In almost all cases, PM is a terminal diagnosis.

When cells lose their attachment and are forced to suspend in a fluid environment, they undergo a detachment-induced apoptosis termed anoikis [5, 6]. Anoikis has been demonstrated to be an important mediator for acinar lumen formation in normal breast tissue, and anoikis resistance has been demonstrated to be critical for mammary tumor formation and metastases [7]. Anoikis resistance is a hallmark of all cancer cells [8, 9] but may be especially important in the biology of PM as the primary mode of tumor dissemination. We and others have previously shown the importance of autophagy to the anoikis resistance phenotype in multiple spheroid cell line models derived from tumor types associated with PM [10]. However, modulation of autophagy has shown limited clinical relevance to date and thus new molecular targets are needed. To this end, we examined temporal changes in the transcriptome during spheroid formation across multiple cell types to identify potential driver signals.

We previously identified BNIP3 as part of a spheroid signature and as an important modulator of autophagy during anoikis resistance [10]. This signature was developed by a comparative analysis of the transcriptome of attached cells and mature spheroids. Recent work has demonstrated that thioredoxin interacting protein (TXNIP) and its binding partner DNA damage response 1 (REDD1) increase in response to stress and stabilize intact autophagy and downregulate TORC signaling [11, 12].

TXNIP has been implicated as both a tumor suppressor and an oncogene and is critical for regulating the redox status in cells through its interaction with thioredoxin (TRX). Elevated levels have been reported to cause cell cycle arrest and apoptosis [13–15]. Reduced levels of TXNIP have been associated with increased mitogenic signaling including increases in TORC signaling [16, 17]. Conversely, a recent report on lung cancer patients demonstrated that TXNIP was hypoxia inducible in lung cancer cell lines and that patients with high levels of TXNIP had significantly shorter PFS which was maintained in a multivariate analysis.

In this paper, we identify TXNIP alterations and resultant TORC signaling changes as central to PM through the use of a novel clustering methodology that identifies outlier genes from gene expression data. This led us to hypothesize that TXNIP acts to downregulate TORC signaling to promote spheroid development in an *in vitro* model system of attachment-independent growth. We demonstrate that TXNIP acts in a context-dependent fashion as both a growth regulator and a growth promoter. We further demonstrate that abrogation of TXNIP-associated TORC signaling is able to prevent PM *in vitro* and in xenograft models.

## RESULTS

### Time series transcriptomic analysis identifies TXNIP as a key gene associated with spheroid development

**Figure 1:**
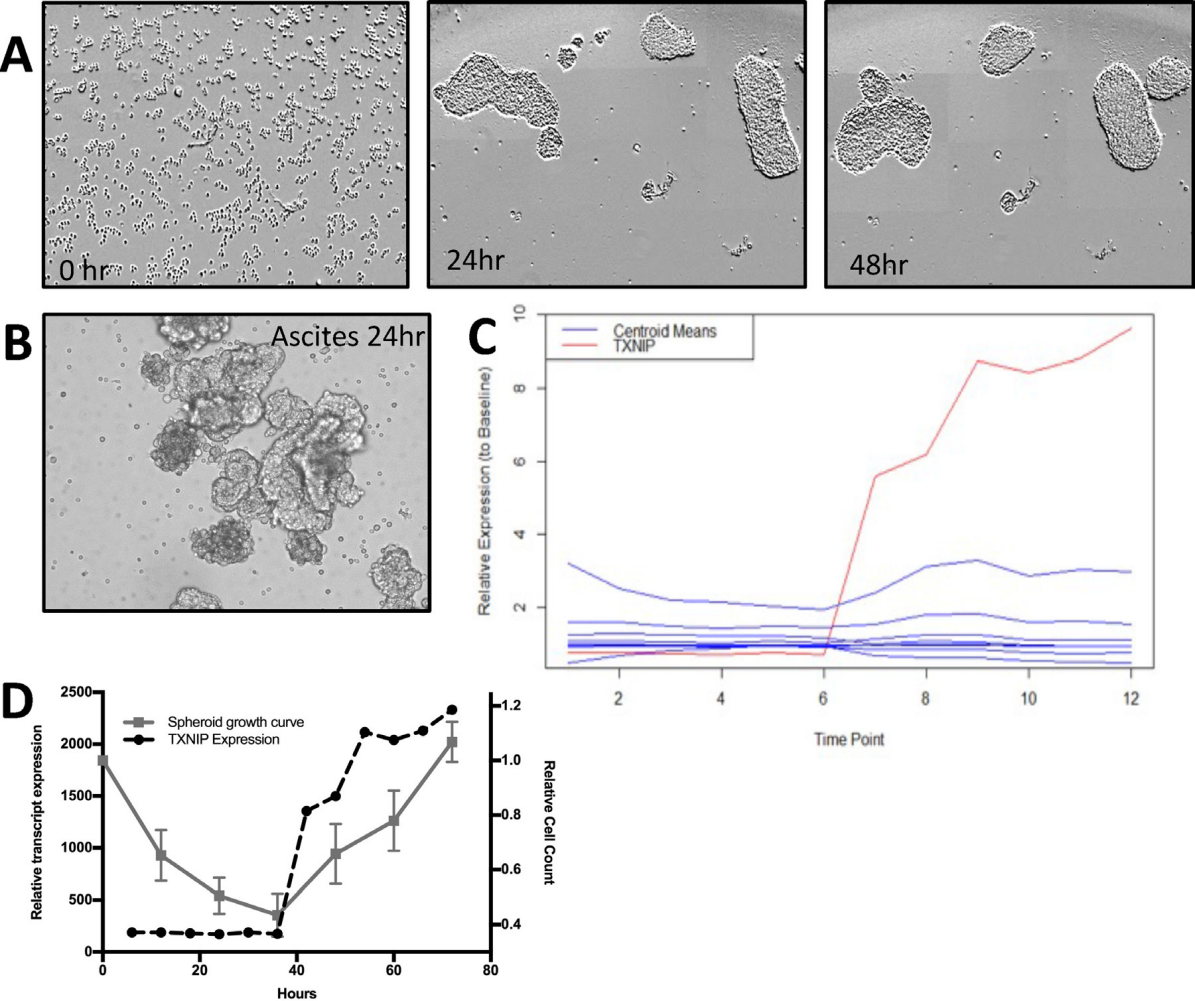
(**A** and **B**) HEYA8 cells were plated in polyHEMA coated 96-well plates and allowed to form spheroids over the indicated times. As a comparison, ovarian cancer cells from ascites were also plated on polyHEMA and allowed to grow for 24 hrs. (**C**) Temporal outlier analysis of HEYA8 cell line. Centroid means identified through Gaussian mixtures model (blue lines) and TXNIP RNA expression of HEYA8 cell line (red line). (**D**) Temporal comparison of cell count from HEYA8 spheroid formation (left y-axis) and TXNIP RNA expression (right y-axis).

To assess the transcriptomic alterations in spheroid development, five cell lines HEYA8 (ovary), PANC1 (pancreas), YOU (malignant peritoneal mesothelioma), TC71 (sarcoma), and CHLA10 (sarcoma) cells representing tumors that can spread via PM were grown in attachment-free conditions on polyHEMA coated plates. Each cell line was used to produce free-floating tumor spheroids (Figure 1A). These spheroids showed a similar size and morphology to patient-derived spheroids (PDS) isolated from ascites of ovarian cancer patients (Figure 1B) [10]. Spheroids were collected every 6 hours for RNA isolation.

This transcriptomic time series data was subsequently analyzed using a novel outlier analysis to identify potential driver genes. In brief, we used Gaussian mixtures model to identify clusters of temporally expressed genes with Mahalonobis distance analysis to identify potentially biologically relevant outliers (Supplementary Methods). A key outlier gene measured in all five cell lines analyzed was TXNIP. In particular, TXNIP was the top outlier in our ovarian cancer cell line (HEYA8, Supplementary Table 1, Figure 1C). TXNIP RNA levels closely tracked with spheroid formation (Figure 1D). We therefore hypothesized that TXNIP may play a key role in PM and spheroid development.

### Elevated TXNIP RNA is associated with poor survival in late stage ovarian cancer, but is associated with improved survival in earlier stage disease

**Figure 2:**
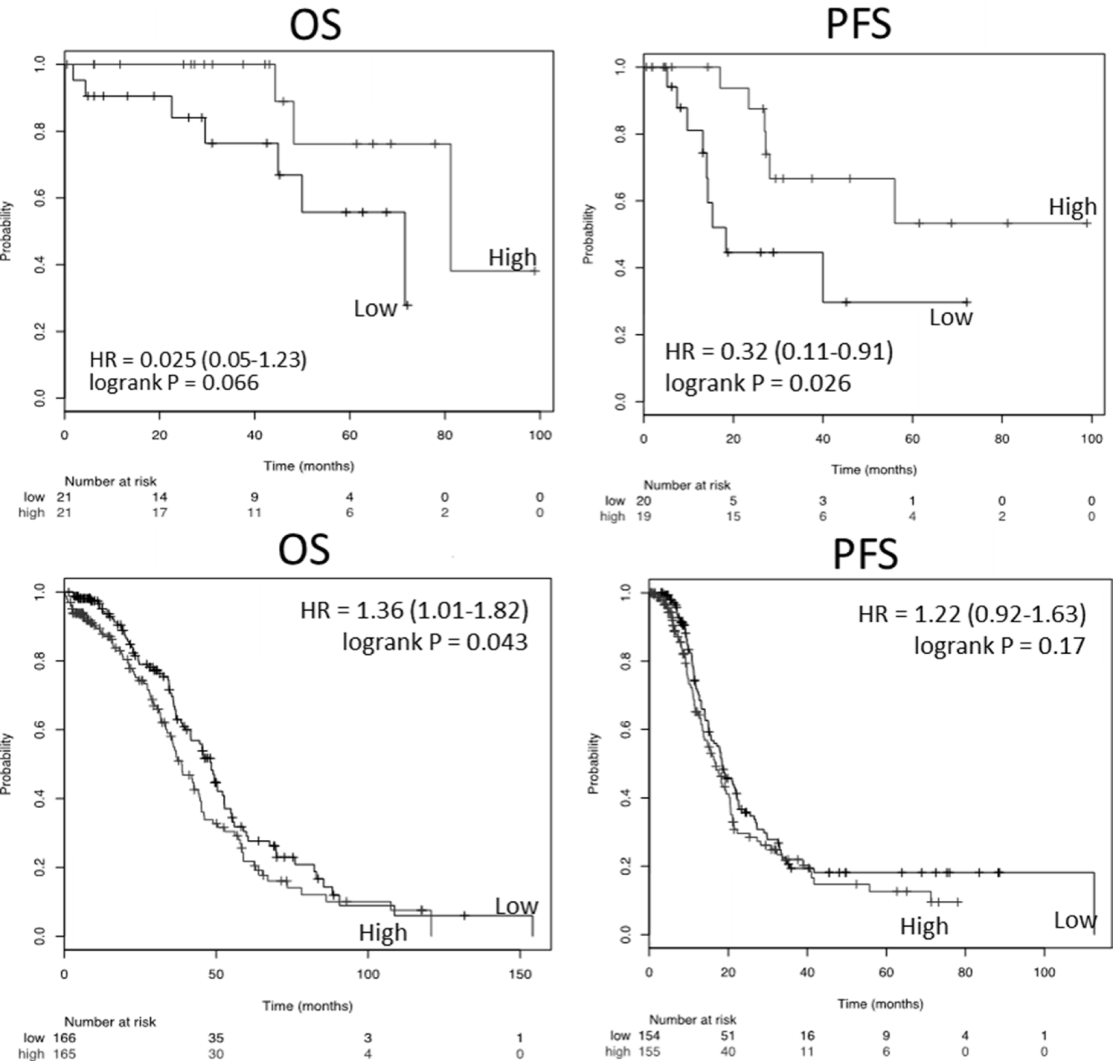
Kaplan–Meier analysis of survival as a function of TXNIP expression in the ovarian cancer TCGA Top panels represent Stage 1 and 2 and bottom panels stage 3 and 4 ovarian cancer samples. Left column represents overall survival (OS) and right column progression free survival (PFS).

Given the conflicting reported significance for TXNIP in various tumors and the increased expression identified in our outlier analysis of *in vitro* spheroids, we examined if TXNIP was prognostic in clinical ovarian cancer patient samples. We chose ovarian cancer as a model system since staging is driven by peritoneal spread of the tumor. We used the TCGA dataset to determine the prognostic significance of TXNIP expression in early and late stage ovarian cancer patients (Figure 2). When TXNIP expression value was split at the median, stage 1 & 2 patients in the low TXNIP group demonstrated a shorter PFS (log-rank *p* = 0.026) and OS (log-rank *p* = 0.066). Conversely, in patients with stage 3 and 4 ovarian cancer, the low TXNIP group exhibited a survival advantage in terms of OS (log-rank *p* = 0.043). PFS for this group trended toward statistical significance (log-rank *p* = 0.17).

### TXNIP levels rise in patient derived spheroids

**Figure 3:**
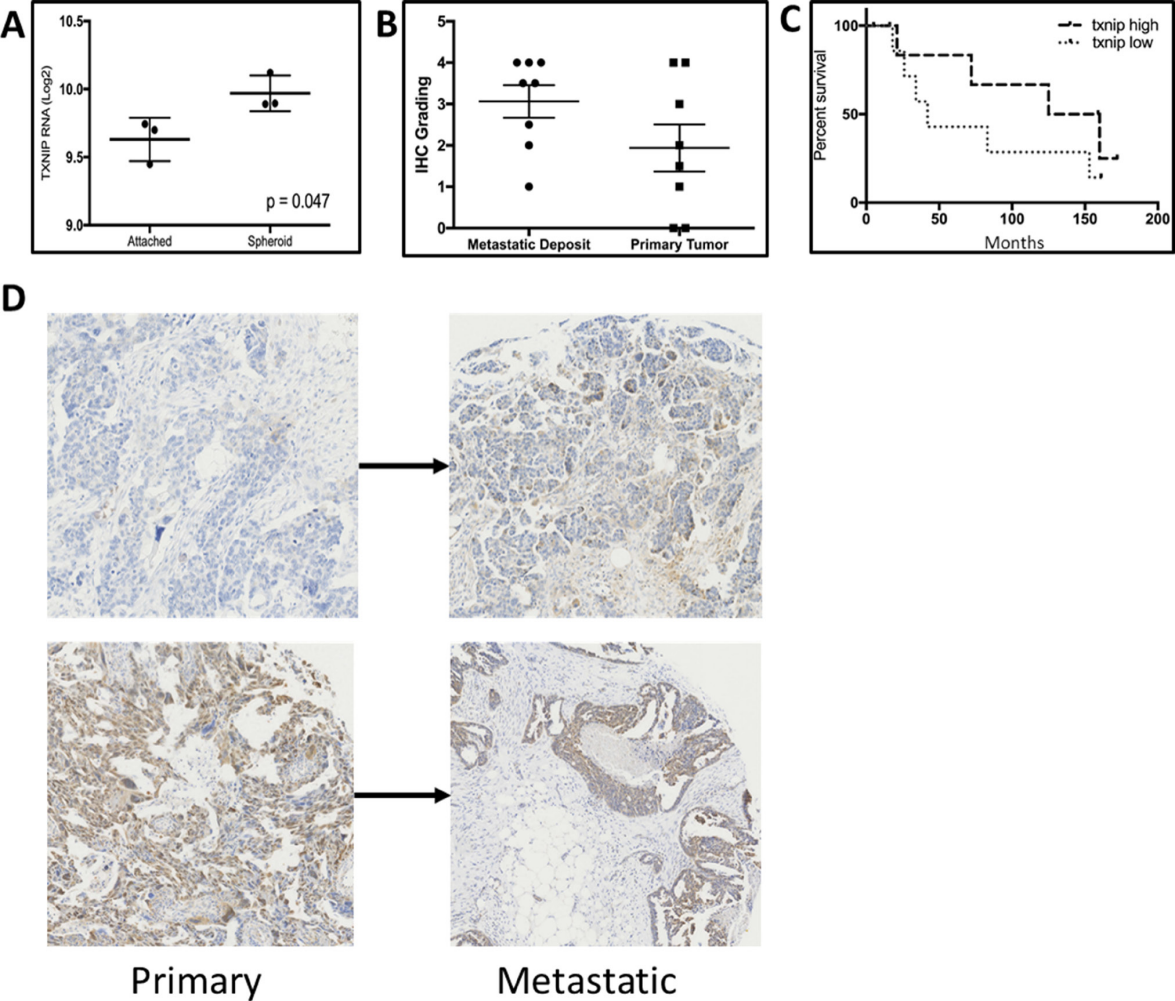
(**A**) TXNIP RNA isolated from tumor cells from primary ovarian cancer ascites grown as attached or under attachment independent conditions as spheroids. (**B**) TXNIP protein levels quantified by IHC from matched primary and metastatic tumor deposits from ovarian cancer patients. (**C**) Kaplan–Meier analysis of PFS for Stage 1 and 2 ovarian cancer patients as a function of TXNIP protein expression. (**D**) Representative IHC of paired primary and metastatic deposits from patients with ovarian cancer demonstrating a wide variation in staining intensity.

In a set of three ovarian cancer patients for whom we have collected ascites (IRB-approved protocol OSU: 13142), we isolated and purified tumor cells from the ascites by placing them in attached conditions and passaging twice as previously described [19]. PDS were subsequently derived by placing the cells in attachment-free conditions. PDS were allowed to mature for 72 hours. We then compared TXNIP RNA levels between the attached patient-derived cells and the free-floating PDS. There was a 25% increase in TXNIP levels (Figure 3A, *p* = 0.047).

We then examined an ovarian cancer tissue microarray with 8 matched primary and metastatic deposits. Elevated levels of TXNIP were noted in metastatic deposits, as compared to matched primary tumors (Figure 3B, *p* = 0.038 and Figure 3D). However, similar to the data analyzed from TCGA, low levels of TXNIP in the primary tumor correlated to worse overall survival in the TMA cohort of 22 patients with stage 1 and 2 disease (Figure 3C, *p* = 0.2) although the results do not reach statistical significance due to a small sample size.

### TXNIP knockdown activates the TORC pathway and decreases tumor growth in cell culture but improves the tumors ability to form spheroids

**Figure 4:**
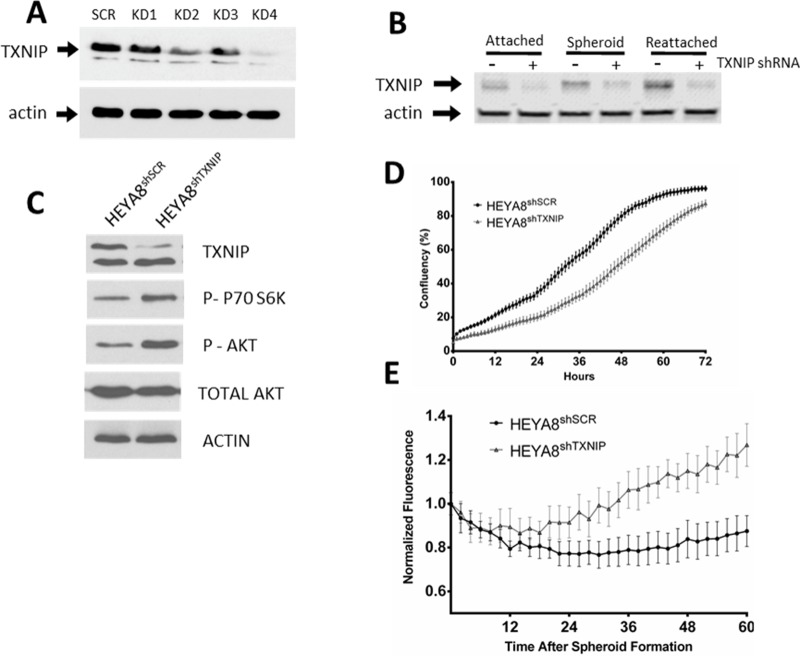
(**A** and **B**) WB of HEYA8 cell line with scrambled vector or TXNIP KD as attached, spheroid or reattached samples. (**C**) TXNIP KD results in TORC activation. (**D**) Growth of HEYA8 cell line with either scrambled vector or TXNIP KD under attached conditions (**E**) or under attachment free conditions on polyHEMA coated plates.

To determine a possible mechanism for this observed dichotomy in patient samples, we then examined the role of TXNIP in our *in vitro* spheroid model. We generated stable knockdowns of TXNIP in the HEYA8 ovarian cancer cell line (HEYA8^shTXNIP^) with an independent GFP-fluorescent tag (Figure 4A). TXNIP levels increased in HEYA8 scrambled control (HEYA8^shSCR^) cells but not in HEYA8^shTXNIP^ when grown in attachment-free conditions (Figure 4B). Using live-cell imaging, we quantified the attached growth kinetics of the HEYA8^shTXNIP^ versus the HEYA8^shSCR^ line. We saw that knockdown of TXNIP inhibited growth of attached HEYA8 cells (Figure 4D). However, under attachment-free conditions, the HEYA8^shTXNIP^ cells demonstrated enhanced spheroid growth after spheroid formation which led to significantly increased spheroid survival over the course of 36 hours. (*p* < 0.001 for all data points beyond 2 hrs, Figure 4E). Given the previously published effects of the TXNIP/REDD1 complex on TORC activation, we next examined the effect of TXNIP knockdown on TORC activation in our spheroid model. We noted that targets of both TORC1 (p-S6K) and TORC2 (pAKT) were up-regulated in the HEYA8^shTXNIP^ (Figure 4C).

### TORC 1/2 inhibition abrogates TXNIP knockdown effects on spheroid formation and prevents PM in ovarian orthotopic PM models

**Figure 5:**
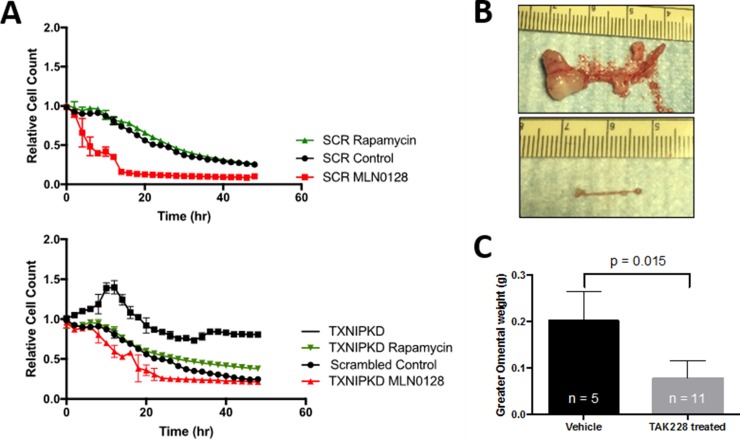
(**A**) In ovarian cancer *in vitro* models, the TORC1/2 inhibitor TAK228 reduces spheroid growth in scrambled and TXNIP knockdown cell lines back to scrambled control levels while rapamycin causes little change within the first 48 hrs in scrambled cell lines however does significantly reduce growth in TXNIP knockdown cell lines. (**B**) In a mouse peritoneal models of ovarian cancer (IP injection of HEYA8 cells), TAK228 when administered 24 hours after inoculation dramatically prevented widespread dissemination of ovarian cancer implants (bottom) as compared to control (top). (**C**) This effect was also seen in TAK228's ability to prevent the increase in the greater omental weight secondary to carcinomatosis (*p* = 0.015).

To evaluate the effects of TORC pathway inhibition on HEYA8^shTXNIP^we used an ATP-competitive inhibitor of both TORC1 and TORC2 complexes, TAK-228, which is currently being evaluated in clinical trials. We have previously shown that rapamycin, a TORC1 complex inhibitor, enhances spheroid formation and growth when counting total surviving cells(10). In HEYA8^shSCR^, Rapamycin showed no measurable differenc in florescence signal during live cell imaging while TAK228 significantly inhibited spheroid growth. The addition of TAK228 to HEYA8^shTXNIP^ *in vitro* reverted spheroid culture dynamics to lower than scrambled control levels while rapamycin inhibited growth of HEYA8^shTXNIP^ spheroids, they still grew slightly faster than HEYA8^shSCR^ (Figure 5A). In a mouse peritoneal model of ovarian cancer, TAK228 dramatically prevented widespread dissemination of ovarian cancer implants (Figure 5B, bottom) as compared to control (Figure 5B, top). All control mice developed PM with noticeable metastatic deposits on the visceral and parietal peritoneal surfaces and increased weight. In mice who received TAK228, no widespread peritoneal metastases were seen and they had significantly decreased greater omental weight (*p* = 0.015, Figure 5C).

## DISCUSSION

Peritoneal carcinomatosis remains an important source of morbidity and mortality for multiple tumor types, particularly that of ovarian cancer. Anoikis resistance is an important functional characteristic of cancer cells when they form spheroids in liquid environments after losing attachment-related signaling. Understanding the molecular aspects of anoikis resistance and spheroid dynamics will lead to improved therapies for this debilitating condition.

One difficulty with understanding anoikis resistance and spheroid growth is the temporally dynamic nature of the system. We and others have previously noted the importance of autophagy [10, 20], EMT-like transitions [21], altered integrin signaling [22], miRNA alterations [23], and the emergence of a stem cell state [24, 25] to anoikis resistance. While these pathways are not completely independent of one another, one's observations may depend on the timing of the examination. In this work, we analyzed anoikis resistance and spheroid growth rates in temporal fashion looking for transcripts that may control various aspects of PM.

We used an *in vitro* model of spheroid formation that recapitulates what we believe occurs in PM. Instead of focusing on clustered families of transcripts, we hypothesized those transcripts that do not fit within the overall shape of a specific temporal cluster, i.e. outliers, may prove to be unique in regulating this process.

High-throughput temporal sequencing data present several challenges in terms of statistical analysis, primarily with respect to dimensionality, complex time-varying covariation patterns, and a high degree of variability in baseline expression. The analysis framework we utilized, based on the Gaussian Mixture Model, utilizes these covariation patterns, scaled to large numbers of observations, and maintains a high degree of biological interpretability. Our method accounts for many of the complexities present in the data, while balancing the need for the result to be a straightforward list of hypotheses for further study. In fact, this method is sufficiently flexible to accomplish this goal for a variety of related experimental designs (e.g. different numbers of time points, different gene panels, etc.) and maintain these characteristics. Our framework is thus an attractive option for analyses where the end goal is hypothesis generation and prioritization.

In our analysis, TXNIP had the largest Mahalonobis distance from the cluster centroid in an ovarian cancer cell line and was highly ranked in all other cell lines examined. TXNIP has previously been shown to act as both a tumor suppressor and as an oncogene, depending on the context. It has also been associated with modulation of TORC activation, cell cycle arrest, mitogenic signaling and can also be upregulated by hypoxia. The duality of this gene was intriguing in our dataset. We have shown that knockdown of TXNIP decreases the growth rate of cells in attached 2D culture and enhances their anoikis resistance and spheroid formation. We validated this finding in a clinical tissue microarray that showed increased TXNIP in metastatic deposits compared to primary tumors. We also examined the effect of TXNIP on survival in the TCGA dataset. In early stage cancers without diffuse peritoneal dissemination, low TXNIP transcript was associated with poorer survival.

**Figure 6:**
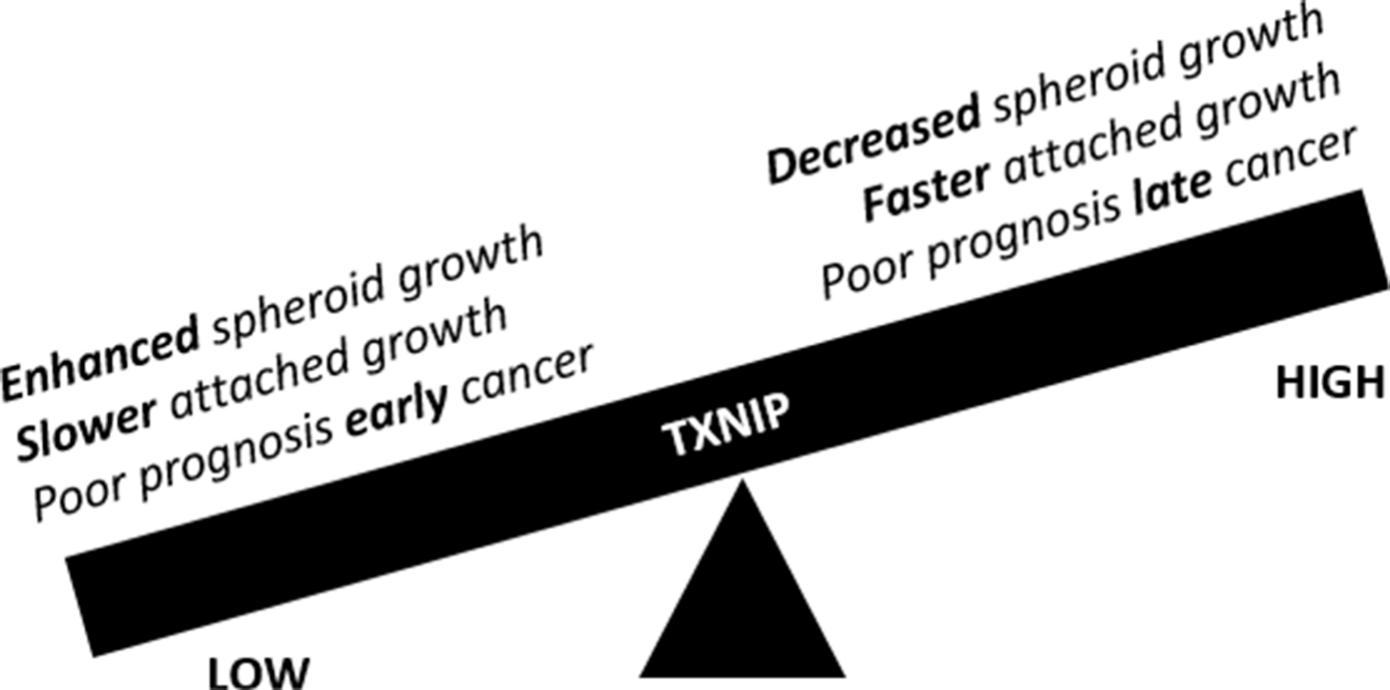
Schematic representation of the duality of TXNIP function as it pertains to peritoneal carcinomatosis/sarcomatosis

This data supports a model where TXNIP enhances growth of attached cells, but inhibits formation and growth of tumor spheroids. This model argues that patients' tumors with low TXNIP may be more likely to form spheroids and therefore be associated with shorter survival due to peritoneal dissemination. However, in patients with diffuse peritoneal disease at diagnosis, high levels of TXNIP, which correlate with faster growth in a 2D model, may drive the mortality associated with this disease (Figure 6). Intriguingly, lowered TXNIP is associated with TORC1/2 activation and thus may serve as a targetable pathway. Here we introduce the use of TAK228 as a therapeutic that may be prevent PM. We demonstrate that it abrogates the effects of low TXNIP on spheroid formation. We also demonstrate that in a mouse orthotopic ovarian cancer model, TAK228 prevents PM when tumor cells are directly injected into the peritoneum.

Our data would agree with the clinical benefit of extensive surgical cytoreduction in patients with disseminated peritoneal carcinomatosis. In these patients, the elevated TXNIP leads to faster attached growth but slower spheroid development and growth. Therefore, debulking the majority of the dividing fraction may impact survival when there is minimal immediate risk for further peritoneal seeding.

PM remains a morbid complication from any number of primary malignancies. At the current time, understanding the molecular basis for PM is limited by relevant model systems that can be queried at multiple time points easily. Here we describe a temporal analysis of an *in vitro* PM model system to describe a novel bimodal gene, TXNIP, which helps provide insight into the molecular switches that drive a tumor to peritoneal dissemination. The effects of downregulated TXNIP on PM potentially can be overcome by the use of TORC1/2 inhibition. Clinical trials studying TORC1/2 inhibitors should consider examining PM as a potential endpoint.

## MATERIALS AND METHODS

### Cell culture

TC71 and CHLA9 were obtained from the Children's Oncology Group (COG). YOU and HEYA8 cell lines were gracious gifts from Raffit Hassan, MD and Elise Kohn, MD, respectively. PANC1 cell line was purchased from ATCC. All cell lines except for TC71 were grown in RPMI-1640 supplemented with 1% penicillin/streptomycin and 10% fetal bovine serum. TC71 required the addition of 1× ITS as per COG specification sheet. For analysis of TXNIP modulation, four TXNIP stable shRNA constructs were generated in HEYA8 cell lines (HEYA8:Scrambled and three independent HEYA8:TXNIP Knockdowns) using OriGene shRNA containing a GFP tag and a puromycin selection marker. Each transfected cell line was selected and maintained in RPMI-1640 supplemented with 1% penicillin/streptomycin, 10% fetal bovine serum, and 5 ug/mL puromycin and a final glucose concentration of 110 mg/dL. No cells were used beyond 10 passages. For drug treatments, TAK-228 (MLN0128) was obtained from SelleckChem (Cat# S2811) and rapamycin was obtained from Sigma-Aldrich (cat# 37094).

### Three dimensional anchorage-independent model

Cells from early passage (<10 from thaw) were seeded in triplicates at 570,000 cells/well in a six-well plate pre-coated with poly-2-hydroxyethyl methacrylate (Poly-HEMA, Sigma cat# P3932). Cells were grown in identical culture media as described above. Culture medium with spheroids were collected and centrifuged @ 500 rpm for 5′ prior to further analysis.

### Immunoblotting

TXNIP antibody was purchased from Cell Signaling (Cat#14715). Cells were lysed using a modified RIPA buffer and gentle vortexing. Samples were normalized to total protein concentration after quantification using a Direct Detect infrared spectrometer (EMD Millipore). TXNIP was quantified using either LiCOR Odyssey system or ECL chemiluminescence. For LiCOR analysis, near-infrared fluorophore secondary antibodies for both the TXNIP and actin antibodies were obtained from Licor. Gels were multiplexed using the red channel for actin loading control and the green channel for TXNIP. Blots were scanned using Licor's Odyssey CLx and images were prepared using Image Studio Lite 5.0. For ECL analysis, anti-Rabbit IgG, HRP-linked secondary antibody was purchased from Cell Signaling (Cat#7073). PVDF membranes were purchased from Biorad (Cat#1620177) and transferred using Biorad Trans-blot^®^ Turbo™ blotting system. Perkin Elmer Western Lightning Plus-ECL, Enhanced Chemiluminescence Substrate (Cat#NEL104001EA) was applied. Blots were developed using GeneMate Blue Autoradiography Film (Cat#F-9024-8×10). Film was scanned using CanonScan LiDE 120 and images were prepared using Image Studio Lite 5.0.

### Live cell, long term, fluorescence microscopy

Spheroids were imaged by seeding GFP-tagged cell lines in eight replicate wells of a 96-well (Greiner CELLSTAR) polyHema coated plate at a density of 16,000 cells per well. Immediately following seeding, plates were transferred to an IncuCyte Zoom (Essen Bioscience) imaging system housed in a temperature and humidity controlled incubator at 5% CO_2_. The IncuCyte software was set to take both brightfield and GFP channel images every hour for 72 hours on “whole well mode” using a 4× Nikon objective. The IncuCyte software (v. 2015A) performed analysis following automatic large image stitching from the tiled images. Each whole well image in the 72 hour time course was thresholded using the GFP channel due to its high signal-to-noise ratio. Pixels registering above 50 GCU (Green Calibration Units) were masked, counted, and reported as a total green object area for each image. All channels were overlaid (brightfield, GFP, green object mask) and time-lapse videos of each well were created. These videos were used to check the accuracy of the GFP channel masks with respect to the brightfield channel. Multiple metrics were generated based on the analysis of segmented images for each time point in the time course. These included: aggregate area of all spheroids in well, total number of spheroids per well, and average size of a spheroid for each well. The data was exported from the Zoom 2015A software, and graphs of the data were analyzed using GraphPad Prism 7.0.

For attached cells, each GFP-tagged cell line was seeded in eight replicate wells of a 96-well (Greiner CELLSTAR) plate at a density of 4,000 cells per well. Immediately following seeding, plates were transferred to the IncuCyte Zoom as listed above. The IncuCyte software was set to take both brightfield images every hour for 72 hours using a 10× Nikon objective. Confluency was analyzed using the brightfield channel by the IncuCyte software. The data was exported from the Zoom 2015A software, and graphs of the data were generated using GraphPad Prism 7.0.

### Timecourse transcriptome analysis

HEYA8 cells were plated under low attachment (polyHema) conditions. Samples were isolated at six hour intervals starting from time of detachment to 72 hours. Total RNA from triplicate cell culture pellets was isolated using the Qiagen RNeasy kit as per manufacturer's directions. RNA quality was confirmed using Nanodrop. RNA was then profiled using a single Illumina HT-12 bead array to avoid batch effect. Raw data was processed using Illumina GenomeStudio and then analyzed using the Outlier Analysis Method as outlined in the Supplementary Methods (Supplementary Table 1). In brief, RNA transcriptomic data was normalized to the time zero point to provide intracohort normalization. A Gaussian Mixtures Model was then applied to the data to define relevant clusters. For genes in the clusters we used the Mahalonobis distance to ascertain its Assigned Centroid Distance (ASC) that is a measure of its conformity to the cluster. The resultant dataset are genes ranked by the ASC. All analyses were performed in R and available for download.

### Retrospective analysis of TCGA data

Data from The Cancer Genome Atlas (TCGA) were accessed using KM-plotter (http://www.kmplot.com), and survival curves were generated using median values of TXNIP (affy id: 201010_s_at) expression to split patient groups as indicated [18]. Only optimally debulked patients were included in the stage 3 & 4 overall survival and progression-free survival plots.

### Tissue microarray

Sections (4–6 μm thick) of formalin-fixed paraffin embedded tissue arrays containing ovarian tumors at various stages were purchased from US Biomax Inc. (Rockville, MD). Slides were baked at 65°C for one hour and immunostaining was performed on the fully automated Bond RX autostaining system (Leica Biosystems, Buffalo Grove, IL). Briefly, heat-induced antigen retrieval was done using ER1 (citrate buffer) for 20 minutes, slides were stained with a rabbit monoclonal antibody to TXNIP (Cell Signaling Cat#14715) at a 1:200 dilution for 30 minutes and the Bond Polymer Refine (DAB) detection system (Leica Biosystems, Buffalo Grove, IL) was used. A pathologist specializing in gynecological pathology read and scored the expression level (1–4+) of various tumors on stained slides, blinded to outcomes data. Survival analysis was performed using Kaplan–Meier estimation using the Graphpad PRISM 6.0 software.

### Mouse xenograft studies

Athymic nude (NCr-nu/nu) female mice, outbred, 7–8 weeks old, were acquired from the athymic nude mouse colony maintained by the Target Validation Shared Resource at the Ohio State University Cancer Center (OSUCCC); the original breeders (strain #553 and #554) for the colony were received from the NCI-Frederick. These female mice were injected with 1 × 10^6^ of HEYA8 ovarian cancer cells intraperitoneal in a 500 ul volume of magnesium chloride and calcium chloride-free phosphate-buffered saline. Approximately 24 hours post-injection of tumor cells, either drug (TAK-228) or placebo treatment was started. TAK-228 (MLN0128) from SelleckChem (Cat# S2811) was formulated in 5% polyvinylpyrrolidone (PVP, Sigma), 15% *N*-methylpyrrolidone (NMP, Sigma) and 80% water; this formulation also served as placebo. Mice were administered TAK-228 or placebo by oral gavage, once daily, 1 mg/kg/day, five days a week (M-F) for three and half weeks, and euthanized within 2–4 hours after the last dose was administered. Body weight was measured daily. Upon necropsy, visceral and parietal peritoneal surfaces were closely observed for tumor deposits; greater omentum was isolated and weighed.

## SUPPLEMENTARY MATERIALS TABLE





## Abbreviations

PM: peritoneal metastasis
TCGA: The Cancer Genome Atlas
TMA: tissue microarray
TXNIP: thioredoxin interacting protein
TRX: thioredoxin
COG: Children's Oncology Group
PDS: patient-derived spheroids
