# Supplementary material for: Loss of TXNIP enhances peritoneal metastasis and can be abrogated by dual TORC1/2 inhibition
Source: Oncotarget. 2018 Nov 2;9(86):35676–86. doi: 10.18632/oncotarget.26281 (PMC6235015; doi:10.18632/oncotarget.26281)
Supplement: Supplementary file 1 [file oncotarget-09-35676-s001.pdf]

Loss of TXNIP enhances peritoneal metastasis and can be abrogated by dual TORC1/2 inhibitio

SUPPLEMENTARY MATERIALS

Outlier analysis methodology motivation

Most time dependent analyses of large datasets begin with partitioning the data into groups with a similar temporal morphology (e.g. clustering techniques). While there are broad classes of regulatory dynamics to which most genes will belong, there exists a small subset of genes that do not fit neatly into these general temporal motifs. We hypothesized that these so-called outliers will play important biological roles in attachment independence.

Methodology

Let $0 < t_1 < \cdots < t_m$ denote the measurement times at which cells are collected and RNA is extracted. Assume that $0 = t_0$ denotes the attached state. Let denote the total number of unique genes sequenced in the sample and for $i = 1, \ldots, G$, let $(y_{i,0}, y_{i,1}, \ldots, y_{i,m})'$ denote the vector of post-processed (i.e. quantile normalized across the time domain) absolute transcription measurements collected at times $(t_0, t_1, \ldots, t_m)$. We assume that all measurements are present (i.e. have been measured directly or imputed). Our method clusters genes according to the relative temporal transcription measurement, hence, define

$$z'_i = \left(z_{i,1}, \ldots, z_{i,m}\right) = y_{i,0}^{-1}\left(y_{i,1}, \ldots, y_{i,m}\right), \quad i = 1, \ldots, G$$

as the relative temporal transcription of gene i. Hence, our clustering ignores the baseline expression values by normalizing them to 1 in the attached state (t = 0). This removes preference based on absolute transcription levels. The assumed statistical model is that for each i the vector $(z_{i,1}, \ldots, z_{i,m})$ is an independent realization from an m-dimensional Gaussian Mixture Model (GMM) with $k \geq 1$ components and mixture probabilities $(\pi_1, \ldots, \pi_k)$ such that $\sum_{j=1} \pi_j = 1$. Let $\psi\left(Z_1, \ldots, Z_m \mid \mu, \Sigma\right)$ denote the m-dimensional Gaussian probability density function with mean $\mu = (\mu_1, \ldots, \mu_m)$ and covariance matrix $\Sigma \in \mathbb{R}^{mxm}$. Let $\theta = (\pi_1, \ldots, \pi_k, \mu_1, \ldots, \mu_k, \Sigma_1, \ldots \Sigma_k)$ denote the entire parameterization of the underlying GMM. Then equivalently we assume that:

$$f(Z_1, \ldots Z_m | \theta) = \sum_j \pi_j \psi(Z_1, \ldots, Z_m | \mu_j, \Sigma_j), \quad \sum_j \pi_j = 1.$$

Conditional on the observed data $(z'_1, \ldots, z'_G)$ the free parameters in θ are estimated via Maximum Likelihood and the number of mixture components (k) selected via optimality of the Bayes Information Criteria (BIC). Let $\hat{\theta}$ denote the estimated parameters conditional on the selection of k' components. Each gene i is then assigned to a cluster $j = 1, \ldots, k'$ according to the maximum conditional probability

$$cl(i) = max_j \hat{\pi}_j \psi(z'_i \mid \hat{\mu}_j, \hat{\Sigma}_j), \quad i = 1, \ldots G.$$

This specifies the cluster membership, each cluster corresponding to a broad post-detachment transcriptional motif, for each gene that was measured. The hypothesis generation algorithm (Figure 1) proposes that "outlier" genes are those that do not fit well with the typical trajectory in any given cluster. We consider a notion of "distance" between an observation and its assigned cluster in terms of the Mahalanobis Distance metric. This distance metric is intuitively attractive because it accounts for different covariance properties between different motifs. Thus, for gene we define its Assigned Centroid Distance (ASC).

$$ASC_i = \left(z'_i - \hat{\mu}_{cl(i)}\right)' \widehat{\Sigma_{cl(i)}} \left(z'_i - \hat{\mu}_{cl(i)}\right)$$

Outlier genes are therefore those genes with the highest ASC values. In particular, higher ASC values necessarily indicate a worse correspondence with the most probable motif for a given gene. Under the scientific hypothesis that driver genes of the observed biological phenomenon (spheroid proliferation) are those with abhorrent transcriptional patterns after time 0, these genes may be identified as those with high ASC values. Thus, this algorithm models the data and creates a hypothesis prioritization scheme based on the observed data to be verified in further lab experimentation.

Supplementary Table 1: Results of an outlier analysis of temporal transcriptomic data during HEYA8 spheroid formation. See Supplementary_Table_1
